# Recent Progress in the Neoadjuvant Treatment Strategy for Locally Advanced Esophageal Cancer

**DOI:** 10.3390/cancers13205162

**Published:** 2021-10-14

**Authors:** Sicong Hou, Ziyin Pan, Xin Hao, Qinglei Hang, Yanbing Ding

**Affiliations:** 1 Department of Gastroenterology, Affiliated Hospital of Yangzhou University, Yangzhou University, Yangzhou 225000, China; shou@yzu.edu.cn; 2 Department of Clinical Medicine, Medical College, Yangzhou University, Yangzhou 225001, China; pan_ziyin@126.com (Z.P.); yzdxhx@163.com (X.H.); 3 Department of Experimental Radiation Oncology, The University of Texas MD Anderson Cancer Center, Houston, TX 77030, USA

**Keywords:** esophageal cancer, neoadjuvant therapy, chemotherapy, chemoradiotherapy, immunotherapy, esophagectomy, active surveillance

## Abstract

**Simple Summary:**

Neoadjuvant therapy is recommended as standard care for patients with locally advanced resectable esophageal cancer. Neoadjuvant chemotherapy (nCT) and chemoradiotherapy (nCRT) have convincingly been shown to improve the survival rate compared with surgery alone based on the results of several randomized clinical trials. Immunotherapy has become a new research direction in the field of EC research due to its great curative effects. However, controversies still remain in regard to identifying the most appropriate combination of nCT, nCRT, immunotherapy, and surgery, optimizing more effective neoadjuvant treatment protocols and surveillance strategies. This review comprehensively summarizes the research progress and describes and discusses the outcomes, pros, and cons of current trials. We believe our work has great academic value and will be of great help for researchers to understand the domestic and foreign research status in the field of neoadjuvant therapy in EC.

**Abstract:**

Neoadjuvant therapies, primarily chemotherapy and chemoradiotherapy, are able to improve the overall survival (OS) in patients with locally advanced resectable esophageal cancer (EC) based on the results of several randomized clinical trials. The advantage of neoadjuvant therapy is chiefly attributed to the decreased risk of local–regional recurrence and distant metastasis. Thus, it has been recommended as standard treatment for patients with resectable EC. However, several fundamental problems remain. First, the combination of neoadjuvant chemotherapy (nCT), neoadjuvant chemoradiotherapy (nCRT), and surgery for EC patients with different histological types remain controversial. Furthermore, to reduce the toxicity of preoperative chemotherapy and the risk of complications caused by preoperative radiation therapy, the treatment protocols of nCT and nCRT still need to be investigated and optimized by prospective trials. Moreover, for patients with complete clinical response following neoadjuvant therapy, it is worth ascertaining whether a “watch and wait” surveillance plus surgery-as-needed policy is more favorable, as well as, in addition to preoperative chemoradiotherapy, whether immunotherapy, especially when combined with the traditional neoadjuvant therapy regimens, brings new prospects for EC treatment. In this review, we summarize the recent insights into the research progress and existing problems of neoadjuvant therapy for locally advanced resectable EC.

## 1. Introduction

Esophageal cancer (EC) is the seventh most commonly diagnosed cancer and the sixth-leading cause of cancer-related death globally according to the statistics in 2020 from the American Cancer Society [1]. Even though esophagectomy is the cornerstone of treatment for locally advanced EC, the loco-regional and distant recurrences still bother nearly half of the patients after surgery [2]. To improve the clinical outcomes, neoadjuvant therapies have been introduced to the curative treatment. The MAGIC and OEO2 trials solidified the role of neoadjuvant chemotherapy (nCT), while the CROSS trial laid the foundation of neoadjuvant chemoradiotherapy (nCRT) for resectable EC [3,4,5]. Although both nCT and nCRT showed the overall survival (OS) benefit compared to surgery alone, current available evidence has not yet supported a clear advantage of nCRT over nCT [6,7,8]. The clinical outcome to different neoadjuvant therapy was also associated with the histological subtypes [9,10]. Despite nCRT and nCT exhibiting a high tumor response to patients with squamous cell carcinoma (SCC) and adenocarcinoma (AC), the nCRT group showed higher sensitivity and significant improvement of survival in SCC patients, while the tumor response was not translated to survival advantages in AC patients [9,11,12,13]. In addition to the efficiency, the treatment protocols of nCT and nCRT also need to consider the toxicity and complications risk. Nowadays, immune-based approaches have shown great potential in EC treatment, which has resulted in a better curative effect when combined with nCT or nCRT regimens [10,14]. Thus, the optimal treatment remains to be determined. In this review, we summarized of the recent insights into the research progress and existing problems of neoadjuvant therapy for EC.

## 2. Neoadjuvant Chemotherapy

### 2.1. Neoadjuvant Chemotherapy Versus Surgery Alone

Chemotherapy works locally and systematically by downgrading the primary tumor to increase the chance of radical resection and elimination of (subclinical) micrometastases, thereby reducing the risk of distant metastases [15]. The safety and efficacy of nCT have gradually been recognized in the field of EC research. The addition of nCT to the treatment regimen for esophageal and gastro-esophageal junction (GEJ) carcinoma was recommended mainly based on the results of several large randomized clinical trials (RCTs) [16,17]. We have made a summary of several pivotal trials in Table 1.

As early as 2002, a large randomized controlled multicenter study was conducted by England Medical Research Council (MRC) with a sample of 802 patients with resectable EC (nCT: 400 cases; surgery alone: 402 cases) [18]. The chemotherapy used in the nCT group consisted of two courses of concurrent administration of cisplatin/5-fluorouracil (CDDP/5-FU, CF). The results showed that nCT significantly enhanced the R0 resection rate (60% vs. 54%), increased the median survival time (16.8 months vs. 13.3 months), and prolonged the 2-year survival rate (43 % vs. 34%) compared to surgery alone. Moreover, patients with SCC and AC had the same risk ratio for treatment outcomes, indicating that the benefit was similar for both histological types. In 2009, a long-term update of the MRC trial called the UK OEO2-trial was conducted, which randomly categorized 802 patients with AC and SCC from 1992 to 1999 into two 4-day cycles of CF (cisplatin: 80 mg/m^2^, fluorouracil: 1000 mg/m^2^) and immediate surgery alone. It was the first trial to identify a significant OS benefit in nCT group compared with surgery alone. Both histological subgroups showed a survival benefit, with a 5-year OS of 23% for AC and 26% for SCC (*p* for interaction = 0.81). The OS benefit can be attributed to the large sample size of the trial and the significant improvement in R0 resections in the nCT group (60% vs. 54%). However, this study found no difference in distant metastasis rates between the two groups, suggesting a comparatively modest systematic benefit of this chemotherapy regimen [5]. The USA RTOG-trial 8911 enrolled 440 patients, and the nCT regimen turned to three cycles of cisplatin (100 mg/m^2^) and continuous infusion of fluorouracil (1000 mg/m^2^) for four days, followed by surgery. Inconsistent with the results of the OEO2-trial, the RTOG trial showed no improvement in local control and OS rate. A possible explanation may be due to the toxicity of the RTOG-trial’s nCT regimen related to a larger amount of cisplatin [22]. Thus, the dosage of cisplatin in CF regimen needs follow-up research to optimize. Although over ten years have passed, the MRC-OEO2 trial still offers great value. Hale et al. [23] collected the digital H/E stained pre-treatment biopsy slides from 281 patients in the MRC-OEO2 trial (141 treated with surgery alone and 140 treated with surgery after nCT). The study investigated the predictive effect of the proportion of tumor cells per tumor area (PoT) measured in pre-treatment biopsies on the treatment benefit of nCT. The results showed a non-linear relationship between PoT and survival, and only patients with PoT between 40% and 70% gained significant benefit from nCT, suggesting for the first time that PoT may be clinically used as a biomarker for patients’ treatment stratification. Further research should focus on improving the prediction model through detailed quantitative morphology and molecular characterization of intratumoral substrate to better understand the underlying biological process, ultimately enhancing EC treatment stratification. Similarly, in another series of original research, Sundar’s team, for the first time continued to conduct DNA methylation analysis of 229 surgically resected specimens from AC patients in the OEO2-trial to identify an epigenetic signature that can serve as a predictive biomarker for the benefit of CF regimen. It is expected to be used for risk stratification and biomarker selection in future AC studies [24]. 

To further improve survival outcomes, several large RCTs have studied a combination of neoadjuvant and adjuvant chemotherapy, i.e., perioperative chemotherapy. As for the MAGIC trial, 503 patients from 1994 to 2002 were randomized into perioperative chemotherapy and surgery alone. These patients were diagnosed with resectable gastric adenocarcinoma, GEJ, and lower esophageal adenocarcinoma. The chemotherapy regimens included epirubicin, cisplatin, and 5-FU (ECF) for three preoperative and postoperative cycles. The results showed that perioperative chemotherapy significantly increased the 3- and 5-year OS from 31% to 44% and 23% to 36.3%, and decreased distant metastases from 37% to 24%, respectively. Preoperative chemotherapy reduced the tumor size in all patients but had limited effects on R0 resection rates [4]. However, since only 25% of participants in the trial had GEJ or lower EAC, the results of this study cannot be extrapolated to all patients with EC without dispute. To cope with this problem, the FNCLCC/FFCD trial randomized 224 patients (more than 70% of all tumors located in the lower esophagus or the GEJ). This trial found a significant OS benefit (38% vs. 24%) in the perioperative CF group compared to surgery alone, as well as an 8% reduction in distant recurrence rates, parallel to results in the MAGIC trial. Furthermore, the FNCLCC/FFCD trial exhibited a significantly improved R0 resection rate in the nCT group compared to surgery alone (84% vs. 74%) [19]. Last but not least, the JCOG9907 trial in 2012 confirmed the benefits of using perioperative chemotherapy using CF regimen as a standard approach in resectable stage II/III SCC [21].

In recent years, the combined chemotherapy using docetaxel, cisplatin, and 5-FU (DCF) has been consistently proven to bring more favorable outcomes in EC. Ojima et al. [25] reported that among patients with clinical stage II/III SCC, a divided-dose DCF regimen yielded a high frequency of pathological response. Hara et al. [26] found that the DCF regimen had strong antitumor activity and was proven to be safe and tolerable for patients with clinical stage II/III SCC. In another prospective multicenter phase I/II study, Satake et al. [27] reported positive oncologic outcomes, as well as bearable post-operative complications and excellent OS performance after using neoadjuvant DCF protocols. As a result, in order to gain more insight into the relative efficacy of DCF regimen, Fiteni’s research team evaluated the efficacy of DCF as perioperative chemotherapy versus surgery alone in a large multicenter comparison cohort of patients with resectable GEA [20]. The trial identified 789 patients who underwent surgery only and 62 patients who received at least one cycle of DCF regimen (docetaxel 75 mg/m^2^ on day 1, cisplatin 75 mg/m^2^ on day 1, 5-FU 750 mg/m^2^/day on continuous perfusion for 5 days) every 3 weeks. In contrast with the surgery group, the chemotherapy group had a better survival rate, higher 3-year OS rate and pathological complete response (pCR), but the improvement of R0 resection rate was not statistically significant. More extensive randomized phase III trials are still needed in the future to explore the potential survival benefit of docetaxel in the perioperative period of resectable GEA. The latest Korean PRODIGY trial and the German NEO-FLOT trial are under way to see whether an updated combination regimen for nCT can bring superior clinical efficacy.

### 2.2. Progression and Optimization in the Treatment Protocols of nCT

In Japan, esophagectomy followed by two courses of neoadjuvant chemotherapy with CF is considered to be the standard strategy for the treatment of patients with advanced or recurrent EC. Although the results from the subgroup analysis of the JCOG9907 trial showed that CF regimens were able to benefit patients with clinical stage III SCC, patients’ survival was limited, indicating the need for different effective regimens [21,28]. We have made comparisons between some different treatment protocols for nCTs and results are shown in Table 2. OEO5 is an open-label phase-III RCT which enrolled 897 patients with resectable AC between 2005 and 2011 who were randomized to four cycles of nCT with epirubicin, cisplatin, and capecitabine (ECX) or two cycles of CF regimen. The results suggested that compared with the CF group, more patients in the ECX group had a good pathological response to chemotherapy (Mandard tumor regression grade 1 or 2) and showed a significant down-staging effect with ypT0 or ypT1 or ypN0. However, no increase was found in patient survival. The median overall survival and disease-free survival expectancy were calculated to be 23.4 months (95% CI 20.6–26.3) and 11.6 months (95% CI 8.9–13.3) in CF group versus 26.1 months (95% CI 22.5–29.7) and 14.4 months (95% CI 11.7–16.5) in ECX group. In addition, there was no obvious 3-year OS benefit either (ECX group: 42% vs. CF group: 39%) [29]. The FLOT-4 trial, presented at the American Society of Clinical Oncology meeting, received significant attention [30]. Patients with stomach or GEJAC underwent four preoperative and four postoperative cycles of FLOT (fluorouracil, leucovorin, oxaliplatin, docetaxel), and another group of patients received three preoperative and postoperative cycles of ECF/ECX. The results showed that the pCR rate significantly increased by 10% in FLOT group compared with ECF/ECX group (16% vs. 6%). After a median follow-up of 43 months, FLOT significantly improved the median and 5-year OS rates, from 35 months to 50 months and from 36% to 45%, respectively. FLOT is now considered as one of the standards of care for AC patients [31]. 

Although the nCT regimen DCF is of particular value for EC treatment, related research and trials are still ongoing. For resectable advanced SCC, the OGSG1003 Phase II trial randomly assigned 162 patients to group DCF and ACF (adriamycin, cisplatin and 5-FU) to make a comparison between the two chemotherapy regimens [32]. The primary endpoint was recursion-free survival (RFS); the secondary endpoints were OS, R0 resection rate, histo-pathological response, and postoperative complications. The final results indicated that DCF had a higher 2-year RFS than ACF (64.1% vs. 42.9%), while the R0 resection rate was similar (96.2% vs. 95.9%, *p* = 0.93). In addition, DCF had significantly better histological findings on major tumor (*p* < 0.0001) and a much earlier pathological T stage (*p* = 0.008). The frequency of recurrence after operation was higher in patients treated with ACF compared with DCF (*p* = 0.008), indicating a better safety from DCF regimen. It is worth noting that no significant difference between DCF and ACF was found in OS; a possible reason is that the follow-up period was too short (34.5 months). Therefore, a follow-up study was conducted to assess the long-term outcomes and analyze the primary endpoint, RFS, as well as secondary endpoints such as OS and recurrence patterns [33]. Consistent with the preliminary results, this study confirmed a better 5-year RFS for DCF (59.9% vs. 40.7%). Encouragingly, the following subgroup analysis showed that the OS of the DCF group was significantly better than that of the ACF group in patients with advanced clinical T (cT3 or cT4) stage and N (cN2 or cN3) stage EC. In contrast, there was no difference in OS between the two groups in patients with early clinical T (cT1 or cT2) stage and N (cN0 or cN1) stage. Based on the data presented above, DCF can be seen as a potential nCT candidate for patients with clinical stage III and IV EC; as for stage I and II, a less toxic regimen should be considered. Given the fact that the DCF group had a remarkable higher control rate for local and distant lesions as well as a lower postoperative local recurrence rate, it is reasonable to speculate DCF regimen as a promising candidate for neoadjuvant therapy for resectable SCC. However, further prospective studies are still required to address the topic due to the limitation of the sample scale of the present trial. The JCOG1109 trial is currently underway in Japan, whose results will prove evidence with regard to the most suitable and effective preoperative treatment for SCC. 

Despite the advantages of DCF, severe grade 3/4 adverse events, especially grade 3 or 4 neutropenia in 66.2–78.2% patients and febrile neutropenia in 14.5–22.9% patients, have drawn great attention. Due to the relatively high toxicity of DCF, some clinical studies have focused on adjusting the dose and frequency of the regimen in the hope of maintaining efficacy while reducing toxicity [36,37]. To integrate the modified DCF regimen (mDCF) into non-metastatic esophageal cancer (nMEC), a retrospective review of 30 patients diagnosed with nMEC and treated with mDCF regimen between 2008 and 2017 was performed [34]. The mDCF regimen refers to a kind of modified weekly DCF: docetaxel 40 mg/m^2^ on day 1, cisplatin 40 mg/m^2^ on day 1, 5-FU 2000 mg/m^2^ on days 1–2, leucovorin 400 mg/m^2^ on day 1. The results have shown that mDCF is not only effective in nMEC and AC patients (64.9% and 44.5% of OS at 3 and 5 years), but also reduces the incidence of severe neutropenia to only 13%. In addition to the impact on AC, the mDCF regimen showed promising results for local advanced SCC, with a 71.4% 2-year OS and a significant reduction in the incidence of grade 3/4 hematologic toxic events (43% for leukopenia and 14% for neutropenia) compared to the maternal DCF regimen. It is worth mentioning that elderly patients and patients with comorbidities account for a large proportion of EC patients, and the possibility of DCF causing adverse events increases. Therefore, the bDCF, a refined regimen in which docetaxel was divided and administered biweekly, was introduced to reduce serious adverse events in these frail patients. Akiyama et al. [35] investigated the feasibility and efficacy of esophagectomy after bDCF treatment in 59 patients with advanced SCC. The trial found that bDCF regimen brought a higher clinical response rate and a lower incidence of grade 3 or 4 neutropenia compared with DCF. Therefore, for patients with advanced SCC, the bDCF nCT regimen turned out to be feasible and safer in the perioperative period without reducing the efficacy of the conventional DCF regimen. 

Apart from adjusting the dosage and frequency of medication, the renewal of drugs as well as the continuous optimization of different drug combinations have also become the critical research issues of nCT for EC. Nab-paclitaxel (Nab-PC), a new generation formulation of paclitaxel, has been used with cisplatin and has shown excellent performance in terms of the down-staging rate, R0 resection, and pCR rate [38]. S-1, developed in Japan, is a kind of oral fluorouracil alternative to infusional 5-FU and is considered effective and safe in treating patients with advanced SCC [15,39]. The cisplatin analogue nedaplatin has shown to be potentially active against SCC with reduced toxicity compared to cisplatin. The biweekly triple chemotherapy with docetaxel, 5-FU, and nedaplatin (UDON) has been proved to have good antitumor activity and tolerability. UDON has been evaluated as a first-line treatment for patients with advanced or recurrent SCC in Japan [40].

## 3. Neoadjuvant Chemoradiotherapy

In most Western countries, nCRT followed by esophagectomy has become a standard of care for patients with locally advanced resectable EC [41]. The largest multicenter RCT of nCRT compared with surgery alone was the Dutch CROSS (Chemoradiotherapy for Oesophageal Cancer Followed by Surgery Study) trial initiated in 2004. The CROSS trial randomized 366 patients between nCRT that consisted of carboplatin and paclitaxel for 5 weeks and concurrent radiotherapy (41.4 Gy in 23 fractions, 5 days per week) followed by surgery versus surgery alone. The multimodal therapy resulted in a pCR rate of 29% (49% in SCC, 23% in AC), an R0 resection rate of 92%, and a median survival time of 49.4 months, which were significantly higher than those in the surgery alone group. The long-term follow-up data also showed 3-, 5- and 10-year overall survival benefit, 14%, 13% and 13%, respectively, indicating that the benefit for patients receiving nCRT lasts for at least 10 years [42]. Since then, the CROSS trial has solidified the foundation of nCRT in EC, which was consistent with the results of previous RCTs [43,44,45,46,47,48]. Although it is not difficult to conclude from the CROSS trial that the effects of nCRT on SCC seem larger than AC, it is hard to widely extrapolate this result because of the small fraction of SCC patients. The recently published NEOCRTEC5010 trial randomized 451 patients with SCC to receive either nCRT plus surgery or surgery alone [13]. The results showed that nCRT improved the 5-year survival compared to surgery alone group (59.9% vs. 49.1%, *p* = 0.025), which was more favorable than those of the CROSS trial [13]. Moreover, the R0 resection rate was higher in the nCRT group than surgery alone (98.4% vs. 91.2%, *p* = 0.002) and the pCR rate reached 43.2%. Therefore, the nCRT may be considered as a practical approach for patients with locally advanced SCC. However, the results of the French FFCD9901 trial, which included 195 patients (70% with SCC), showed that no significant difference between the nCRT and surgery arm regarding the R0 resection rate and 3-year survival [46]. Due to there being no benefit in both arms, the trial was stopped. A possible explanation might be that the FFCD9901 trial only included early-stage (fewer node-positive and T3 stage) patients and the perioperative mortality increased in the nCRT group. Therefore, these findings suggested that nCRT was not suitable for patients with an early clinical-stage tumor and offered an important reference to researchers about this treatment approach, although the results of the FFCD9901 trial were negative. The clinical trials mentioned above have been summarized in Table 3. Nevertheless, based on the effectiveness of perioperative chemotherapy for AC, several RCTs are ongoing to evaluate the optimal neoadjuvant therapy in patients with AC. The Neo-AEGIS trial is designed to further compare nCRT with the CROSS regimen versus the perioperative chemotherapy with modified MAGIC regimen (ECF/ECX or EOF/EOX) [49]. Moreover, considering the distinct advantage of the FLOT regimen in perioperative chemotherapy mentioned above [50,51], the phase III randomized ESOPEC trial is developed to compare the FLOT-regimen-based perioperative chemotherapy versus CROSS-regimen-based nCRT [52]. The results of these trials will be bound to provide new evidence for the standard care in patients with locally advanced resectable AC in the near future.

However, with the emerging findings regarding the neoadjuvant interventions, many controversies remain, such as optimizing more effective nCRT protocols and surveillance strategies post-nCRT, and better patient selection for nCRT and surgery. In the following paragraphs, we focus on the research progress addressing these existing problems.

### 3.1. Comparison between the Treatment Protocols of nCRT

Based on the JCOG9204 and JCOG9907 trial results, nCT with CF regimen is a standard treatment for patients with cStage II/III SCC in Japan. In Western countries, the OEO2 RCT was carried out, in which 66% of patients with AC also showed a survival advantage in the neoadjuvant CF regimen group. In comparison, the CROSS trial has been proved to provide the maximum ever seen survival benefits for patients with esophageal or junctional cancer and has defined a new benchmark for the benefits from nRCT. Of note, using carboplatin and paclitaxel instead of the traditional CF regimen is one major innovation in the CROSS trial [2]. However, other trials also cast doubt on treating the CROSS regimen as the standard of care. A retrospective review showed higher rates of pCR and improved recurrence-free and OS in EC patients who completed nRCT with CF regimen compared to the CROSS regimen [54]. In addition, although the FLOT-4 trial revealed comparable 3-year survival in the docetaxel-based triplet regimen group with AC in the subgroup of the CROSS trial, the FLOT regimen is more simplified and less toxic. Therefore, prospective direct comparison RCTs involving direct comparisons are necessary to evaluate the relative merits of different chemotherapy options. 

Another treatment strategy that has been a common concern is the induction of chemotherapy before nCRT and surgery. A multicenter phase II RCT (NCCTG N0849) evaluated the effect of adding induction chemotherapy prior to nCRT in EC patients [55]. Induction chemotherapy consisted of docetaxel (60 mg/m^2^, day1), oxaliplatin (85 mg/m^2^, day1), and capecitabine (625 mg/m^2^, day 1–14) every 21 days for two cycles followed by nCRT with 5FU, oxaliplatin, plus concurrent daily radiation (50.4 Gy in 28 fractions). The primary endpoint of this study was the pCR rate, and secondary endpoints included OS and disease-free survival (DFS). A total of 8 of 28 (28.6%) patients that underwent induction chemotherapy had pCR versus 40.7% of patients that underwent nCRT followed by surgery (*p* = 0.34). Given no statistical differences between the two groups, the trial was terminated, but the patients were followed. However, after a median follow-up of 60.4 months, a separation in OS was unexpectedly observed favoring the patients treated with induction chemotherapy over no induction (3-year survival rates 57.1% vs. 41.7%). Moreover, induction (versus no induction) chemotherapy was associated with longer OS and DFS, particularly among patients with well/moderately differentiated tumors. Consistently, several recent studies also indicated that the intensification of preoperative cytotoxic chemotherapy brings no benefit to unselected patients with non-metastatic EC, indicating that other approaches are supposed to be considered to improve survival outcomes [29,56]. Future prospective evaluation trials should focus on the novel induction therapy (e.g., immunotherapy plus chemotherapy) that can improve OS and risk-stratification based on tumor differentiation by modern strategy (e.g., FDG-PET).

Going back to the aforementioned CROSS trial, another major innovation was the use of a lower neoadjuvant radiation dose (41.4 Gy in 23 fractions) instead of the standard dose (50.4 Gy in 25–28 fractions). To our knowledge, no converse data have exhibited inferior outcomes of 41.4 Gy in the nCRT process. As we know, higher radiation doses produce potentially better tumor control, but also expose patients to increased risks according to the sigmoidal curve for cell kill versus radiation dose in principle of radiation biology. A retrospective study from the USA using the National Cancer Data Base (NCDB) showed that a high neoadjuvant radiation dose is associated with an increased pCR rate (*p* < 0.001) and 30-day mortality, but with no difference in OS [57]. Considering the risk of complications, such as cardiac toxicity and radiation pneumonitis, a relatively lower neoadjuvant dose was recommended. Several groups recently demonstrated that although a lower radiation dose may result in slightly lower pCR rates (not significantly statistical difference) versus a high radiation dose, it did not affect the oncological outcomes [57,58,59]. Moreover, how to regulate the radiation dose for patients who drop out from neoadjuvant to definitive radiation therapy is another issue of concern. Therefore, prospective evaluation is of great value and should be designed with caution.

### 3.2. Active Surveillance in Patient Post-nCRT with Complete Response

Given that a substantial fraction of patients receiving nCRT reached pCR [2,9], it is reasonable to reconsider the necessity of surgical resection in those patients who respond sufficiently to nCRT. An active surveillance approach in which patients achieved a clinically complete response (cCR) after nCRT are subjected to serial clinical investigations; surgery was only offered to patients with loco-regional regrowth/residual disease. Similar approaches have been proved with curative results in several types of malignancy, including rectal, prostate, and head and neck cancer [60,61,62]. Therefore, the feasibility and efficacy of an active surveillance approach has been recently evaluated in patients with resectable EC after the completion of nCRT in different study designs [63,64,65,66,67,68,69]. The Germany Stahl et. al., France FFCD9102, and Korean ESOPRESSO trials are phase-III RCTs aiming to compare the clinical outcomes in complete responders to nCRT in EC. The primary outcome of the European trials is OS, while the Korean trial used DFS as the primary outcome. All published results are summarized in Table 4. Notably, the intervention arm (active surveillance plus surgery as needed) exhibits no relevant difference regarding OS in all three RCTs compared to the control arm (surgery on principle); only the ESOPRESSO trials showed decreased DFS, which might be due to the frequent local recurrence during surveillance [63,64,65,66]. In fact, early identification of resectable local recurrence without simultaneous distant dissemination is more critical than DFS itself. In order to establish the optimal combination of diagnostic techniques for clinical response evaluations (CRE) after nCRT, a prospective preSANO trial was designed and has already been completed at multicenters in the Netherlands [70]. The primary endpoint was the association of clinical response with the final pathological response, as shown by the proportion of tumor regression grade (TRG) 3 or 4 (>10% residual carcinoma in the resection specimen) tumors that went missing during CRE process. The results showed that 31% (95% CI 17–50) of TRG3/4 residual tumors went missing by endoscopy with regular biopsies and fine-needle aspiration (FNA), 10% (95% CI 4–23) went missing by bite-on-bite biopsies and FNA, 28% (95% CI 17–44) went missing by endoscopic ultrasonography (EUS) plus FNA, and 15% (95% CI 7–28) went missing by PET-CT. These findings provided the optimal combination of diagnostic modalities of CRE after nCRT in EC patients, which consisted of EUS, bite-on-bite biopsies, and FNA of suspicious lymph nodes for the detection of locoregional residual disease, with PET-CT for the detection of interval metastases. This combination of diagnostic tests helps to stratify the patients who might benefit from active surveillance 4–6 weeks after nCRT and is now being assessed in a phase III RCT (SANO trial). Apart from the Netherlands SANO trial, France ESOSTRATE and Chinese CELAEC are ongoing RCTs investigating the issue of active surveillance in patients with complete response after nCRT [68,69]. Consistent with the results of complete RCTs, retrospective cohort studies have shown the feasibility and non-inferiority of a non-surgical approach strategy in patients without compromising OS rates [71,72]. Van der Wilk et al. reported that patients with cCR undergoing active surveillance or surgery on principle had a 3-year OS of 77% and 55%, respectively (HR 0.41; 95% CI 0.14–1.20, *p* = 0.104), and an equal distant dissemination rate (28%) [73]. Similar results regarding the median OS in the active surveillance group versus surgery on principle are reported by Furlong et al. (55 months vs. 56 months in elderly patients) and Taketa et al. (57.9 months vs. 50.8 months) [72,74]. 

In conclusion, post-nCRT surveillance and surgery as needed are feasible for patients who reach cCRs without compromising OS. Although the benefit of active surveillance strategy has been investigated via different study designs, the currently available results were based on different protocols of neoadjuvant treatment and surveillance strategies. Therefore, there is an urgent need to perform high-quality RCTs regarding an active surveillance strategy for EC.

## 4. Neoadjuvant Chemotherapy Versus Chemoradiotherapy

As mentioned above, nCT and nCRT have been proved to bring a more significant survival benefit than surgery alone for patients with locally advanced resectable EC. However, according to the previous evidence-based findings and current guidelines, nCRT did not show an advantage over nCT, which means the optimal treatment strategy remains controversial. As shown in Table 5, three RCTs, three RCTs directly compared the outcomes of nCRT versus nCT followed by surgery in EC. In the POET trial, patients with locally advanced EGJAC treated with nCRT showed a significantly higher pCR rate (15.6% vs. 2%) and tumor-free lymph nodes (64.4% vs. 27.7%). In spite of the fact that the nCRT arm showed a large but statistically insignificant trend toward 3-year survival benefit, the long-term results still suggested a superiority in local progression-free survival (PFS) to nCT arm [75,76]. Consistent with the observations in the POET trial, higher tumor response (histological complete response rate and R0/R1 resection rate) was also favored by nCRT arm in both the Neo-Res and Australian trial, which were larger completed multicenter RCTs [11,12,77]. However, paradoxically, such a tumor response was not translated to survival advantages. One possible explanation for the lack of survival benefit based on good tumor response in the nCRT group could be that the extensive lymph node dissection was used (lymph node dissection was practiced in 48% of patients with tumor resection in POET trial, 83% in Neo-Res and 100% in Australian trial). On the other hand, the Neo-Res trial defined pCR as a complete histologic response only in resected tumor tissue; in fact, residual tumor was found in lymph nodes in 10% of patients. In addition, given that the common recurrence pattern of AC was distant metastases, no survival benefit of the Australian trial might be attributed to the small number of AC patients who were diagnosed with stage II tumors and received low-intensity chemotherapy.

A recent meta-analysis of eight RCTs involving 1030 patients with resectable EC was published to provide clinical evidence for comparing nCRT with nCT. This study reported a benefit of nCRT over nCT in OS (HR, 0.78,95% CI 0.62–0.99, *p* = 0.04), 5-year survival rate (RR 1.48, 95% CI 1.06–2.07, *p* = 0.02), pCR (RR 3.74, 95% CI 2.03–6.88, *p* < 0.01) and R0 resection rate (RR 1.13, 95% CI 1.07–1.20, *p* < 0.01), while the benefit was not associated with the risk of 30-day postoperative or in-hospital mortality [78]. For the first time, this study provided high-quality evidence to confirm the survival superiority of the use of nCRT over nCT in resectable EC; however, several limitations exist. It was a retrospective study including limited centers with diverse patient characteristics and therapy approaches, so further prospective studies in boarder populations are necessary.

The currently ongoing RCTs with larger numbers of patients primarily try to answer two questions. The first is whether nCRT has survival advantages over nCT; the second is whether the more appropriate chemoradiotherapy could improve clinical effects and minimize the adverse events. The Neo-AEGIS trial randomized 594 patients with ACs of esophagus or EGJ between pre- and post-operative chemotherapy with MAGIC or FLOT regimen versus nCRT with CROSS regimen [49]. The NeXT trial included a total of 501 patients with SCC who were randomized to nCT arm with CF or DCF regimens and nCRT arm with CF plus 41.4Gy radiation [79]. Moreover, immunotherapy has gained promising clinical benefits in neoadjuvant therapy, suggesting that the personalized combination of immunotherapy and chemoradiotherapy should be further investigated to improve the treatment effects.

## 5. New Dimensions in Neoadjuvant Therapy for EC

### 5.1. Molecular Targeted Therapy Combined with nCT or nCRT

SCC is a deadly disease with a low 5-year survival rate, which requires multidisciplinary treatment. In recent years, targeted sequencing technologies have been developing at a fast speed. As a result, molecularly targeted therapies have been gradually applied to neoadjuvant therapy for EC, but the results varied. 

Epidermal growth factor (EGFR) is an important biomarker for predicting the outcome of SCC treatment. It has been reported that EGFR signaling pathway plays a crucial role in the growth, proliferation, invasion, and apoptosis of EC cells, and upregulated EGFR can be observed in most EC patients [80,81,82,83]. Therefore, the combination of neoadjuvant therapy with anti-EGFR agents may be a potential method to further improve the clinical efficacy. A prospective multicenter phase IB/II trial (SAKK 75/06) harbored the idea that cetuximab in combination with nCRT (DTX/CDDP + 45Gy RT) helped to improve the R0 resection and pCR rate without toxicity for patients with resectable locally advanced EC [84]. However, Ruhstaller et al. [53] continued to conduct a randomized open-label phase III trial (SAKK 75/08), which included a total of 300 patients. Despite the benefit patients have got from targeted therapis in the median PFS and median OS, this trial demonstrated that adding little or no cetuximab to nCRT yielded a higher R0 resection and pCR rate. Similar negative results were also observed in another clinical study. Preoperative cetuximab combination with nCRT (CPT-11/CDDP + 50.4Gy RT) not only exhibited no significant improvement in pCR rates for patients with locally advanced AC but could also lead to severe toxic and side effects [85]. In addition, the survival benefit of cetuximab added to nCRT remained disappointing in the RTOG-0436 and Scope-1 trials [86,87]. As can be seen from these clinical trials, the efficacy of cetuximab in EC is still controversial and even contradictory. 

Nimotuzumab (nimo) is kind of a recombinant humanized IgG monoclonal antibody, which finally inhibits the EGFR signaling pathway by effectively blocking the binding of EGF and transforming growth factor–α (TGFα) [88]. According to a basic in vitro study, nimo promotes EC cells’ radiosensitivity by upregulating the expression of IGFBP-3 [89]. Jing et al. argued that nimo in combination with nCRT appears to show more potential than cetuximab in treating locally advanced ESCC [90]. Nimo improved the disease control rate (DCR) (79.7 vs. 73.9, *p* = 0.04) and significantly prolonged PFS (19.6 months vs. 13.0 months, *p* = 0.02) without causing grade 3 or even more serious toxicity. The cheerful results on the treatment response and survival condition of nimo will be supportive to its further clinical study. Chen et al. enrolled 195 patients with locally advanced thoracic ESCC in a retrospective study to make a comparison between nimo-nCRT, nCRT alone (CF + 40Gy RT), and nCT alone (CF). The results showed that the addition of monoclonal antibodies was safe, the R0 resection rate reached 100%, and the pCR rate reached 41.2%. Compared with nCRT and nCT alone, the R0 resection and pCR rate, respectively, increased by 4.1% and 7.4%, and 8.8% and 26.4% [91]. In another phase I study, Qi et al. [92] concluded that nimo combined with nCT (paclitaxel/carboplatin + 41.4Gy RT) had a favorable anti-cancer effect in locally resectable advanced EC with tolerable toxicities. Although the results from the above works are encouraging, it should be noted that the sample size is still limited, and the results cannot obtain further confirmation. For this reason, a large, multicenter, randomized phase III trial (NCT02409186) is underway to examine the efficacy of nCRT in combination with nimo in locally advanced SCC [93]. With the increasing clinical use of nimo, its drug resistance has gradually been discovered and significantly cut down on the available options for patients with EC. Sun et al. [94] published a case report of a patient who eventually developed nimotuzumab resistance after receiving multidisciplinary treatment with immune checkpoint inhibitors followed by nimo combined with chemotherapy (nab-paclitaxel, TN regimen). They suggested that the underlying mechanism of nimo resistance stems from the activation of the PI3K/AKT/mTOR pathway by discovering PIK3CA mutation and RICTOR amplification in the next-generation sequencing (NGS) assay. POWER was an open-label phase III superiority trial [95]. In this study, Moehler’s research team held the view that another EGFR inhibitor pa-nitumumab was not beneficial to OS in patients with metastatic SCC. Moreover, a prospective analysis of predictive and prognostic serum and tumor tissue biomarkers reaped further insights into EGFR signaling pathways. Contrary to previous studies, the POWER trial indicated that EGFR expression was neither correlated with clinical parameters nor patients’ prognostic outcomes. Last but not least, the team found that panitumumab induced the release of sEGFR, which turned out to be a negative factor triggering worse PFS. Consequently, the role of sEGFR in anti-EGFR therapies needs clearer explanation and further detection. 

In addition to the EGFR signaling pathway, multitudes of studies are carried out to make contributions to the diversity of molecular targeted therapies in EC. The vascular endothelial growth factor (VEGF) inhibitor bevacizumab was added to perioperative ECX chemotherapy in the ST03 trial, but the results were not optimistic enough [96]. Another two phase-II clinical trials centered on human epidermal growth factor receptor 2 (HER2) and investigated the addition of trastuzumab to perioperative treatment. They successfully reached the consensus that neoadjuvant targeted therapy helped more patients to obtain pCR and led to a higher R0 resection rate [97,98]. Present and future randomized trials are trying to combine targeted monoclonal antibodies with classic nCT and nCRT regimens (CROSS, FLOT, et al.) that have been proven to offer clear clinical advantages [17]. The results of these trials are highly expected by everyone.

### 5.2. Immunotherapy with Checkpoint Inhibitors Combined with nCT or nCRT

Antibody-based immunotherapy, adoptive cell therapy, and vaccine-based immunotherapy yielded great fruits in improving outcomes of tumors. Among them, the birth of immune checkpoint inhibitors (ICIs) opened a new era for the treatment of EC. With the increasing related research, the overexpression of programmed cell ligand-1 (PD-1) has been found to be widely involved in the process of immune evasion, which has a lot to do with EC patients’ adverse pathological response and dreadful prognosis [99,100,101]. Nivolumab and pembrolizumab are two main types of PD-1 ICIs, whose mechanism is to block the immunosuppressive action of ligand, which is of great help for the body’s immune system to clear tumor cells from the body. In 2019, pembrolizumab was approved by the FDA as a second-line treatment for PD-1-positive patients [102]. In a multi-cohort Phase IB study (KEYNOTE-028), the overall response rate for 23 PD-1-positive SCC and EGJ patients was 30% and the median duration of response reached 15 months [103]. Furthermore, KEYNOTE-181 aimed to evaluate the anti-tumor activity of pembrolizumab as a second-line therapy compared with chemotherapy alone. This work fully proved prolonged median OS (9.3 vs. 6.7 months) and acceptable toxicity in the mAb group [104]. Nivolumab is human IGG4 ICI preventing anti-tumor T cells’ inactivation. In 2019, Kato et al. [99] implemented the ATTRACTION-3 trial to carry out a final analysis of nivolumab’s curative effect for patients with advanced ESCC refractory or intolerance to the former chemotherapy regimen. They concluded that compared with chemotherapy (paclitaxel or docetaxel), nivolumab possessed a better survival benefit and showed a better safety property. Moreover, Japan has approved nivolumab as a second-line immunotherapy for advanced unresectable or recurrent EC since 2020 [105]. 

This year, Shen et al. [106] administered 28 patients with resectable locally advanced ESCC to evaluate the feasibility and safety of a neoadjuvant treatment protocol of PD-1 ICIs (pembrolizumab: 2 mg/kg, nivolumab: 3 mg, camrelizumab: 200 mg) combined with nCT (albumin paclitaxel + carboplatin). This regimen produced an unprecedentedly high R0 resection and PCR rate. More importantly, researchers had new discoveries. For one thing, after this innovative neoadjuvant regimen, tumors tended to adhere to the surrounding tissues more loosely, which permitted easier resection in surgery. For another, no pseudoprogression was observed in this trial. Despite all of these positive findings, it is still uncertain what the optimum number of neoadjuvant therapy cycles is, and there is an urgent need to identify predictive biomarkers to help scientists examine the effect of immunotherapy precisely with the aim of determining the best subsequent treatment options. To address the problems, Wu et al. [107] investigated the expression of immuno-related molecules and held the view that the expression of the sum of lesion diameter (SLD) was positively associated with the pathological remission rate of EC to a significant extent. Moreover, evidence from the PERFECT-trial suggested that it is promising to explore the IFN-γ signature and baseline combined positivity score (CPS) for PD-L1 as potential biomarkers [108]. These scientific works have exerted a profound impact on future immunotherapy for EC patients. 

Immunotherapy combined with nCRT has been proven to potentiate a synergistic effect. Local radiotherapy increases T-cell infiltration and antigen presentation, sup-porting immune-mediated out-of-field (abscopal) effects [109,110,111]. Achievements of a phase III trial (CheckMate577) have been reported at the 2021 ASCO meeting. This study supports that DFS and distant metastasis-free survival (DMFS) were significantly longer among patients who received nCRT followed by nivolumab [112]. However, a large multi-center or multi-ethnic study is still required to further evaluate the safety and efficacy of nCRT combined with immunotherapy in resectable EC. 

In the end, with the development of immunotherapy, several key questions remain to be taken into consideration. On the one hand, the central premise of cancer immunotherapy is patients’ certain degree of immunity to exert antitumoral properties. Consequently, the efficacy will be greatly reduced for patients with immunologic failure. On the other hand, a great many previous studies indicated that nCT and nCRT regimens could produce toxicity, which mainly leads to immunosuppression. Further studies are supposed to focus on developing more innovative ICIs and exploring how to maximize the therapeutic efficacy of immunotherapy.

## 6. Conclusions

Neoadjuvant therapy is an essential part of multi-modality treatments in patients with EC. It has become a research hotspot due to its high efficacy and diversified combination regimens. Compared with surgery alone, nCT and nCRT consistently improved survival condi-tions for either SCC or AC patients. However, controversies still exist. In this review, we focus on the research progress in investigating innovative chemotherapy regimens to reduce the incidence of serious complications and identify more effective nCRT protocols and post-nCRT active surveillance strategies. In addition, neoadjuvant immunotherapy and targeted therapy have shown excellent efficacy and bright development prospects, which provide more options. A multitude of clinical studies are currently ongoing in search of the best combination therapy. Hence, selecting suitable chemotherapeutic agents and the optimal radiotherapy strategy, screening the target population, and avoiding severe complications and adverse reactions are still important research directions of the future work in the field of EC. We believe that substantial breakthroughs will be made through more and more pro-spective RCTs in the near future.

## Figures and Tables

**Table 1:** Clinical trials of nCT versus surgery alone for EC patients.

Trial	Histologic Subtype	TNM Stage	Intervention	Patients (*n*)	CT	R0 (%)	MST (Months)	OS (%) (1; 2; 3; 4; 5 Years)	Postoperative Morbidity (%)	Postoperative Mortality (%)
MRC 2002 [18]	AC, SCC	NA	nCT→S	400	CF (2 cycles)	60	16.8	-; 43; -; -; -	NA	10
			S	402		54	13.3	-; 34; -; -; -		10
MAGIC 2006 [4]	AC	T1–4, N0–3	nCT→S	250	ECF (3 cycles)	69	NA	-; -; -; -; 36.3	46	5.6
			S	253		66		-; -; -; -; 23	45	5.9
OEO2 2009 [5]	AC, SCC	NA	nCT→S	400	CF (2 cycles)	60	NA	-; -; -; -; 23	NA	NA
			S	402		54		-; -; -; -; 17.1		
FNCLCC/FFCD 2011 [19]	AC	T0–4, N0, N+	nCT→S	113	CF (2 or 3 cycles)	84	NA	-; -; -; -; 38	NA	4.6
			S	111		74		-; -; -; -; 24		4.5
Fiteni et al., 2016 [20]	AC	T0–4, N0–N3	nCT→S	62	DCF (≥1 cycle)	93	57	-; -; 67; -; -	34	3.2
			S	789		85	22	NA	52	2.9
JCOG 9907 2012 [21]	SCC	T2–3, N0–1	nCT→S	164	CF (≥1 cycle)	96	NA	-; -; -; -; 44	NA	NA
			S	166		91		-; -; -; -; 39		

TNM, tumor–node–metastasis; CT, chemotherapy; R0, complete macroscopic and microscopic tumor resection; MST, median survival time; OS, overall survival; nCT, neoadjuvant chemotherapy; S, surgery; SCC, squamous cell carcinoma; AC, adenocarcinoma; NA, not available; CF, cisplatin/5-fluorouracil; ECF, epirubicin/cisplatin/5-fluorouracil; DCF, docetaxel/cisplatin/5-fluorouracil.

**Table 2:** Comparison between different regimens of nCT.

Trial	Histologic Subtype	TNM Stage	Intervention	Patients (*n*)	nCT	R0 (%)	pCR (%)	MST (Months)	OS (%) (1; 2; 3; 4; 5 Years)	Median DFS (Months)	Postoperative Morbidity (%)	Postoperative Mortality (%)
OEO5 2017 [29]	AC	T1–4, N0–1	nCT→S	446	ECX (4 cycles)	66	NA	26.1	-; -; 42; -; -	14.4	62	3
			nCT→S	451	CF (2 cycles)	59		23.4	-; -; 39; -; -	11,6	56	2
FLOT4 2019 [30]	AC	T2–4, N0–3	nCT→S	360	ECF/ECX (3 cycles)	78	6	35	-; 59; 48; -; 36	18	50	3
			nCT→S	356	FLOT (4 cycles)	85	16	50	-; 67; 57 -; 45	30	51	2
OGSG1003 2017 [32] 2020 [33]	SCC	T1–4a, N0–3, M0–1	nCT→S	81	ACF (2 cycles)	95.9	NA	NA	-; 65.4; -; -; 49.4	NA	NA	NA
			nCT→S	81	DCF (2 cycles)	96.2			-; 78.6; -; -; 63.5			
Onitilo et al., 2021 [34]	AC	T1–4, N0–2	nCT→S	23	mDCF (4–6 cycles)	NA	NA	44.4	-; 64.9; -; -;44.5	NA	NA	NA
	SCC			7				76.5	-; 71.4; -; -; 71.4			
Akiyama et al.2018 [35]	SCC	T1–4b, N0–3, M0–1	nCT→S	37	DCF	NA	13.5	NA	NA	NA	32.4	0
			nCT→S	22	bDCF		22.7				45.5	0

DFS, disease-free survival; ECX, epirubicin/cisplatin/capecitabine; ACF, adriamycin/cisplatin/5-FU; FLOT, fluorouracil/leucovorin/oxaliplatin/docetaxel; mDCF, modified DCF; bDCF, biweekly DCF.

**Table 3:** Clinical trials of neoadjuvant chemoradiotherapy versus surgery alone for locally advanced resectable EC.

Trial	Histologic Subtype	TNM Stage	Intervention	Patients (*n*)	CT	RT	R0 (%)	pCR (%)	MST (Months)	OS (%) (1; 2; 3; 4; 5 Years)	Postoperative Mortality (%)
Walsh, et al., 1996 [43]	AC	NA	nCRT→S	58	CF (2 cycles)	40 Gy/15 fractions	NA	25	16	52; 37; 32; -; -	3
			S	55				NA	11	44; 26; 6; -; -	2
CALGB 9781 2008 [45]	AC, SCC	T1–3, N0–1	nCRT→S	30	CF (2 cycles)	50.4 Gy/28 fractions	NA	40	53.8	-; -; -; -; 39	0
			S	26				NA	21.5	-; -; -; -; 16	4.2
CROSS 2012 [9]	AC, SCC	T1, N1 or T2–3, N0–1, M0	nCRT→S	178	TC (5 weeklycycles)	41.4 Gy/23 fractions	92	29	49.4	82; 67; 58; -; 47	4 in hospital
			S	188			69	NA	24.0	70; 50; 44; -; 34	4 in hospital
FFCD 9901 2014 [46]	AC, SCC	T1–2, N0–1, M0 or T3, N0, M0	nCRT→S	98	CF (2 cycles)	45 Gy/25 fractions	93.8	NA	31.8	-; -; 47.5; -; 41.1	11.1 in hospital
			S	97			92.1		41.2	-; -; 53.0; -; 33.8	3.4 in hospital
NEOCRTEC 5010 2018 [13,53]	SCC	T1–4, N1, M0 or T4, N0, M0	nCRT→S	224	VP (2 cycles)	40 Gy/20 fractions	98.4	43.2	100.1	90.0; 75.1; 69.1; -; 59.9	2.2
			S	227			91.2	0	66.5	86.2; 72.5; 58.9; -; 49.1	0.4

RT, radiotherapy; pCR, pathological complete response; nCRT, neoadjuvant chemoradiotherapy; TC, paclitaxel/carboplatin; VP, vinorelbine/cisplatin.

**Table 4:** Summary of randomized trials of surveillance vs. surgery in clinically complete responses after nCRT.

RCTs	Histologic Subtype	TNM Stage	Intervention after CRT	Patients (*n*)	R0 (%)	pCR (%) (S Group)	DFS (%) (2 Years)	MST (Months)	OS (%) (2; 3 Years)	Postoperative Mortality (%)
Stahl, et al., 2005 [63]	SCC	T3–4, N0–1, M0	A	86	82	35	NA	14.9	35.4; 24.4	3.5
			S	86				16.4	39.9; 31.3	12.8
FFCD 9102 2006 [64] &2007 [65]	SCC, AC	T3, N0–1, M0	A	130	NA	NA	NA	19.3	39.8; -	0.8
			S	129				17.7	33.6; -	9.3
ESOPRESSO 2019 [66]	SCC	cT3-T4a, any N, M0 or any T, N+, M0	A	18	50.0	69	42.7	Not reached	Not reached	0
			S	19	92.3		66.7			5.3

TC, paclitaxel/carboplatin; VP, vinorelbine/cisplatin.

**Table 5:** Clinical trials of neoadjuvant chemoradiotherapy versus chemotherapy for locally advanced resectable EC.

RCTs	Histologic Subtype	TNM Stage	Intervention (*n*)	CT	RT	R0 (%)	pCR (%)	PFS (3; 5 Years) (%)	MST (Months)	OS (3; 5 Years) (%)	Postoperative Mortality (%)
Burmeister, et al., 2011 [11]	AC	cT2–3, cN0–1	nCRT→S (39)	CF (2 cycles)	35Gy/15 fractions	84.6	13	NA	32	52; 45	0
			nCT→S (36)			80.5	0		29	49; 36	0
POET 2009 [75] and 2017 [76]	AC	T3–4, Nx, M0	nCRT→S (60)	PLF (2 cycles) +CE (1 cycles)	30Gy/15 fractions	72.0	15.6	NA	30.8	46.7; 39.5	10.2
			nCT→S (59)	PLF(2.5cycles)		69.5	2.0		21.1	26.1; 24.4	3.8
Neo-Res 2016 [77] and 2019 [12]	AC (75%) SCC	T1–3, any N (except T1N0)	nCRT→S (90)	CF (3 cycles)	40Gy/20 fractions	87	28	44; 38.9	31.4	47; 42.2	58
			nCT→S (91)			74	9	44; 33	36	49; 39.6	60

TC, paclitaxel/carboplatin; VP, vinorelbine/cisplatin.
